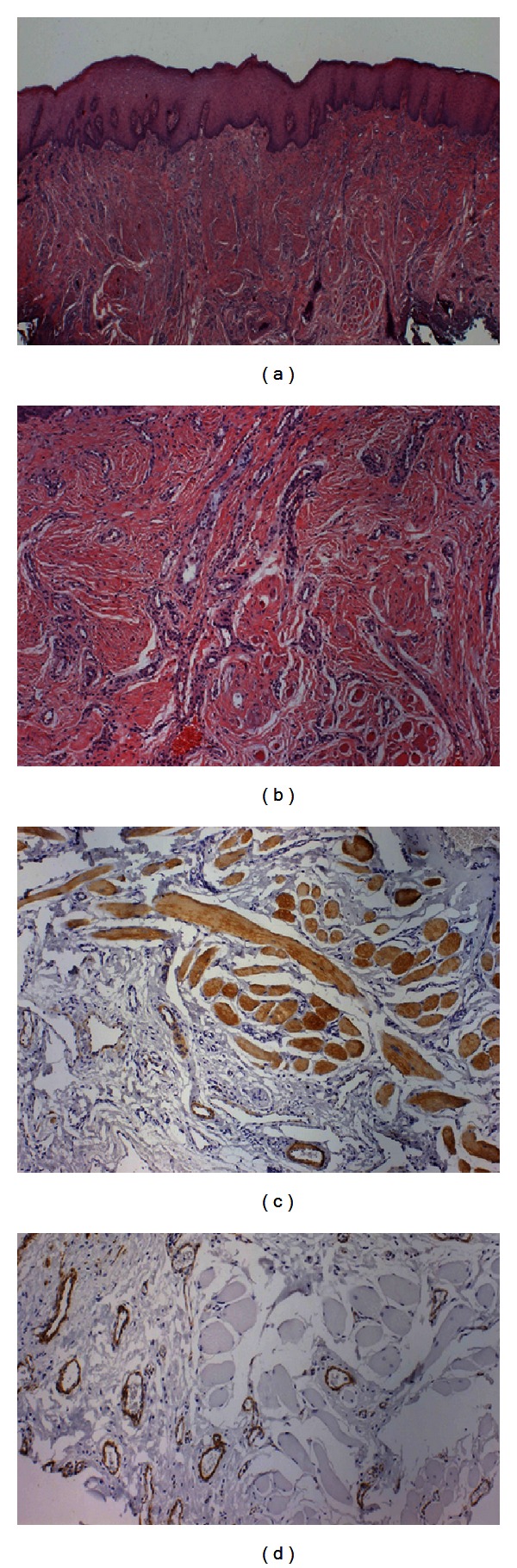# Rhabdomyomatous (Mesenchymal) Hamartoma Presenting as Haemangioma on the Upper Lip: A Case Report with Immunohistochemical Analysis and Treatment with High-Power Lasers

**DOI:** 10.1155/2013/943953

**Published:** 2013-05-28

**Authors:** Aluana Dal Vechio, Edgar Nakajima, Décio Pinto, Luciane Hiramatsu Azevedo, Dante A. Migliari

**Affiliations:** ^1^Department of Stomatology, School of Dentistry, University of São Paulo, 05508-900 São Paulo, SP, Brazil; ^2^Laboratory for Laser in Dentistry (LELO), School of Dentistry, University of São Paulo, 05508-900 São Paulo, SP, Brazil; ^3^Department of Stomatology, School of Dentistry, University of São Paulo, Avenida Professor Lineu Prestes 2227, Cidade Universitária, 05508-900 São Paulo, SP, Brazil

## Abstract

Rhabdomyomatous hamartoma is a rare disease that occurs predominantly in the skin. This paper describes a congenital lesion in a 17-year-old male, who came to our clinic presenting a circumscribed swelling involving the oral mucosa and vermillion border of the upper lip, purplish in color, and blanching under pressure. The patient reported that he had had lesion since his birth. A clinical diagnosis was of congenital haemangioma, and the patient was treated by photocoagulation using diode laser. When the lesion became smaller, by having its blood content reduced, the upper portion of the lesion was sliced off with CO_2_ laser and the tissue was sent for microscopic analysis. Histopathological examination showed an oral mucosa fragment with proliferation of striated muscle bundles admixed with small blood vessels, collagen, and nerve fibres. A supplementary analysis with immunohistochemistry demonstrated positivity for desmin, HHF35, smooth muscle actin, S-100, and CD34. Based on these findings, the lesion was diagnosed as rhabdomyomatous hamartoma. The aesthetic result has been very satisfactory after a 14-month followup.

## 1. Introduction

Rhabdomyomatous hamartoma (RH) is a rare, congenital lesion with only a few reports (approximately 25 cases), most of them affecting the skin, particularly of the face and neck [1, 2]. Lesions are usually described as a solitary papule or nodule, but they can be multiple and, sometimes, associated with congenital abnormalities [1, 2].

This paper describes a case of RH on the upper lip, diagnosed with the aid of immunohistochemical analysis, followed by largely successful treatment with diode and CO_2_ lasers.

## 2. Report

**Figure 1:**
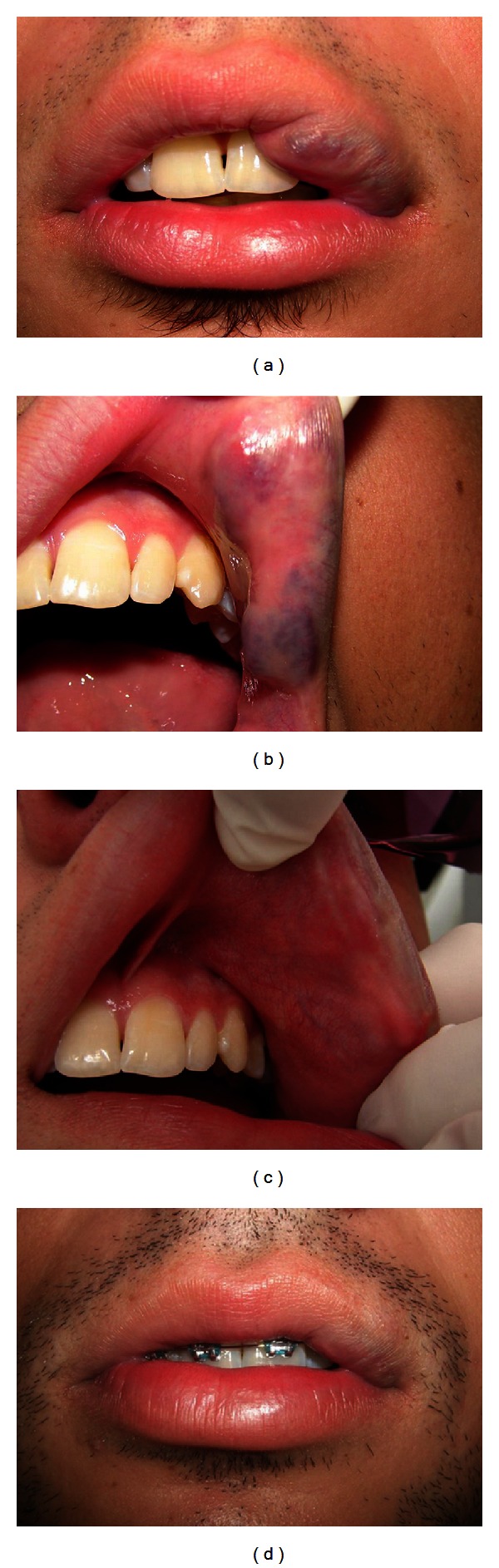


A 17-year-old male came to our clinic for treatment of a circumscribed swelling on the mucosa and vermillion border of the upper lip (Figures 1(a) and 1(b)), 4 cm in length and purplish in color, which blanched under pressure. The lesion had been present since birth. The patient reported that there was occasional bleeding. His main concern, however, was aesthetic. His general health was good.

A clinical diagnosis of congenital haemangioma was made and the patient was treated under local anesthesia by photocoagulation using diode laser at 830 nm with power set at 3.5 W. Irradiation was delivered by means of a flexible quartz fiber that was kept 2-3 mm away from the lesion, in continuous wave mode for 10–20 seconds. This surgical procedure was repeated 3 times at 30-day intervals, thus allowing for recovery of the patient and shrinking of the lesion between sessions. When the lesion became smaller, by having its blood content reduced, the upper portion of the lesion was removed by orienting the CO_2_ laser beam parallel with the mucosa surface and sliced off the top of lesion with one single cut. Subsequently, the tissue was sent for histopathological and immunohistochemical examinations.

The aesthetic result has been very satisfactory after a 14-month followup, with only a discrete swelling appearing on the lip (Figures 1(c) and 1(d)).

The histological examination (HE) revealed oral mucosa covered by squamous epithelium with proliferation of striated muscle bundles admixed with small blood vessels, collagen, and nerve fibres. No cytological atypia, necrosis, or mitosis was observed (Figures 2(a) and 2(b)). Immunohistochemical stain was performed by the streptavidin-peroxidase complex technique. Muscular fibres were positive for desmin and HHF35 (Figure 2(c)); smooth muscles of vessel walls were positive for smooth muscle actin (Figure 2(d)); nerve fibres scattered throughout the lesion were positive for S-100, and blood vessels were positive for CD34. Based on these findings, the lesion was diagnosed as RH.

## 3. Discussion

Hamartoma, a term coined by the Greek (meaning to deviate or miss the goal), is used in the medical literature to describe a disordered arrangement of normal elements forming a tumor-like lesion [2, 3]. Histopathologically, RH is composed of a mixture of mature tissues, haphazardly arranged. 

The clinical form most often reported in the literature of RH is a solitary papule or nodule, occurring particularly on the skin in the region of the head and neck [2]. In the present case, the lesion showed an unusual presentation; besides mimicking a typical congenital haemangioma, it had predominantly an oral involvement, which is an exceedingly rare location, there being only 3 cases on the oral mucosa reported in the literature [2, 4, 5].

The treatment yielded a great improvement of the lesion, and the patient felt very pleased with the aesthetic result, reporting little concern about the remaining slightly bluish-swollen aspect of his left upper lip.

In the present case, the histopathological analysis coupled with immunohistochemical assay was highly instrumental for disclosing the congenital origin of the lesion while the high-power lasers treatment provided a safe and effective resolution, largely successful and without scarring.

## Figures and Tables

**Figure 2:**